# Ecological fitting is a sufficient driver of tight interactions between sunbirds and ornithophilous plants

**DOI:** 10.1002/ece3.5942

**Published:** 2020-02-05

**Authors:** Štěpán Janeček, Kryštof Chmel, Guillermo Uceda Gómez, Petra Janečková, Eliška Chmelová, Zuzana Sejfová, Francis Luma Ewome

**Affiliations:** ^1^ Department of Ecology Faculty of Science Charles University in Prague Praha Czech Republic; ^2^ Biology Centre Czech Academy of Sciences České Budějovice Czech Republic; ^3^ University of South Bohemia České Budějovice Czech Republic; ^4^ Bokwango Buea Cameroon

**Keywords:** bird pollination, co‐evolution, convergent evolution, nectarivore, niche differentiation, trait matching

## Abstract

1. Plant–bird pollination interactions evolved independently on different continents. Specific adaptations can lead to their restriction when potential partners from distant evolutionary trajectories come into contact. Alternatively, these interactions can be enabled by convergent evolution and subsequent ecological fitting.
2. We studied the interactions between New World plants from the genus *Heliconia*, Asian plants of genus *Etlingera* and African sunbirds on a local farm in Cameroon. *Heliconia *spp. evolved together with hummingbirds and *Etlingera* spp. with spiderhunters —an oriental subgroup of the sunbird family.
3. Sunbirds fed on all studied plants and individual plant species were visited by a different sunbird spectrum. We experimentally documented a higher number of germinated pollen grains in sunbird‐visited flowers of *Etlingera* spp. For *Heliconia* spp., this experiment was not successful and pollen tubes were rarely observed, even in hand‐pollinated flowers, where enough pollen was deposited. The analyses of contacts with plant reproductive organs nevertheless confirmed that sunbirds are good pollen vectors for both *Heliconia* and *Etlingera* species.
4. Our study demonstrated a high ecological fit between actors of distinct evolutionary history and the general validity of bird‐pollination syndrome. We moreover show that trait matching and niche differentiation are important ecological processes also in semi‐artificial plant‐pollinator systems.

## Graphical abstract

The interactions between African sunbirds and alien ornithophilous plants from Asia and America were studied at a local farm in Cameroon. We demonstrated a high ecological fit between actors of distinct evolutionary history.

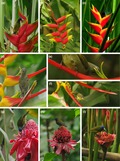

## 1 INTRODUCTION

Evolutionary trajectories and related adaptations of ornithophilous plants and nectarivorous birds differ on individual continents and in different phylogenetic plant and bird lineages (Abrahamczyk, 2019; Fleming & Muchhala, 2008). Considering specialized nectarivores, the three largest groups are hummingbirds (Trochilidae) in the New World, sunbirds, and spiderhunters (Nectariniidae) in Africa, Asia, and Australia and honeyeaters (Meliphagidae) in Australia, New Zealand, New Guinea, and many South Pacific islands (Cheke, Mann, & Allen, 2001; Cronk & Ojeda, 2008; Schuchmann, 1999). Similarly, bird‐visited plants can be found in many families. Flores, Ornelas, Wethington, and Arizmendi (2019) reported 105 plant families that contain plant species visited by hummingbirds, 54 of which they classified as ornithophilous or partly ornithophilous. In Australia, Ford, Paton, and Forde (1979) reported 31 plant families visited by birds. In 15 of these families, not only bird‐visited but also bird‐pollinated species were found. In sunbirds, Cheke et al. (2001) documented the occurrence of food plants in 94 families. For South Africa, Rebelo (1987) reported 30 plant families that contained at least one ornithophilous species.

Many authors have highlighted differences among adaptations in independently evolved bird‐pollination systems as well as similarities which are the consequences of convergent evolution. One of the most famous convergent adaptations of specialized nectarivorous birds is thin bills and tubular tongues which enable them to drink nectar from flowers, even though exact bill and tongue parameters differ among individual bird groups (Paton & Collins, 1989). The common flower properties of ornithophilous plants are defined by the bird‐pollination syndrome. Bird‐pollinated flowers are usually red or orange, without scent and produce a lot of nectar (Cronk & Ojeda, 2008). The most often reported example of specific adaptations is related to the fact that New World hummingbirds usually hover whereas Old World sunbirds perch when feeding (Cronk & Ojeda, 2008; Fleming & Muchhala, 2008; Pyke, 1980). As a consequence, we can find many hummingbird‐pollinated plant species which have their flowers oriented into free space in the New World (Westerkamp, 1990) and different plant adaptations which enable perching of passerine birds in the Old World (Frost & Frost, 1981; de Waal, Anderson, & Barrett, 2012). Nevertheless, this dichotomy is not without exceptions and there are known Old World plants adapted to sunbird hovering (Janeček et al., 2011; Janeček, Bartoš, & Njabo, 2015; Padyšáková & Janeček, 2016). Similarly, as demonstrated by the pollination systems of two *Heliconia* spp., foraging behavior, hovering versus perching, can be species‐specific in hummingbirds (Taylor & White, 2007). Other discussed differences were assumed to be in nectar properties (Baker, Baker, & Hodges, 1998). Johnson and Nicolson (2008) nevertheless demonstrated that different nectars can be more commonly found among plants visited by specialized versus nonspecialized birds than among plants visited by Old World sunbirds versus New World hummingbirds. For the amount of sucrose in nectar, this specialized versus nonspecialized bird dichotomy was confirmed by Abrahamczyk et al. (2017), who, however, showed that sunbird‐pollinated plants have more diverse nectar compositions than hummingbird‐pollinated ones.

The differences and similarities discussed above lead to the question: how do different evolutionary trajectories and convergent evolution processes effect the compatibility of individual pollination systems and what is the ecological fit (Janzen, 1985)? In simple terms: can plants be effectively pollinated by birds and can birds feed on plants when they have different evolutionary histories? The answer to this question is important for understanding the consequences of divergent and convergent evolution processes. Moreover, with the recent acceleration of global environmental changes, it can help us to predict scenarios when native partners are lost thanks to biodiversity degradation and/or new invasive partners occur (Cox & Elmqvist, 2000).

The feeding of nectarivorous birds on non‐native plants has been reported in America (Maruyama et al., 2016), Asia (Ghadiriani, Qashqaei, & Dadras, 2007), Australia (Ford et al., 1979), and Africa (Geerts & Pauw, 2009a). Records of bird pollination of alien plants are much less common. Sunbirds were recorded as pollinators of invasive tobacco *Nicotiana glauca,* which is naturally pollinated by hummingbirds in America (Ollerton et al., 2012). The sunbirds even pollinate it in a similar way to hummingbirds using hovering flight (Geerts & Pauw, 2009a). Also, in the New World it was shown that the ornamental plant *Strelitzia reginae,* which is native to South Africa, can be pollinated by the local common yellowthroat warbler *Geothlypis trichas* (Hoffmann, Fortier, & Hoffmann‐Tsay, 2011). There are, nevertheless, no comparative studies which document how these non‐native interactions are affected by evolutionary history and how they function in more complex systems, where other ecological processes, like niche differentiation, might play a role. In our study, we focus on interactions among African sunbirds and alien ornamental plants on a local farm in Cameroon. These farms produce ornamental plants of the spiderhunter‐pollinated genus *Etlingera* from Asia (Sakai, Kato, & Inoue, 1999; Sakai, Kawakita, Ooi, & Inoue, 2013) and of the hummingbird‐pollinated genus *Heliconia* from America (Linhart, 1973; Temeles & Kress, 2003). Spiderhunters are passerine birds of the genus *Arachnothera*, part of the sunbird family Nectariniidae (Cheke et al., 2001), whereas hummingbirds (family Trochilidae) represent a very distant phylogenetic lineage (Prum et al., 2015). Thus, the farms represent a unique place to study the consequences of specific and convergent adaptations, as well as possible ecological community processes such as niche differentiation. Using this system, we tested the following three scenarios: (a) complete noncompatibility, local sunbirds do not visit any of the plants which evolved on different continents; (b) partial ecological fitting, where birds visit the plants but do not pollinate them (c) full ecological fitting, where birds visit alien plants and pollinate them. Under partial and full ecological fitting scenarios, we expect that sunbirds will show niche differentiation and plants are likely to differ in the spectrum of sunbird visitors. We also decided to determine nectar production and nectar concentration of individual plants to see whether observed patterns can be simply explained by offered rewards.

## 2 MATERIALS AND METHODS

### 2.1 Study site

The study was performed near Bokwango village (4°8′6″N, 9°13′16″E, 940 m a.s.l.), which is administratively part of Buea town (NW Cameroon). The study site was a local farm where the four target species of genus *Heliconia* and three taxa of genus *Etlingera* are commercially cultivated for flower production, within an area of approximately 1 ha.

### 2.2 Plant species

**Figure 1:**
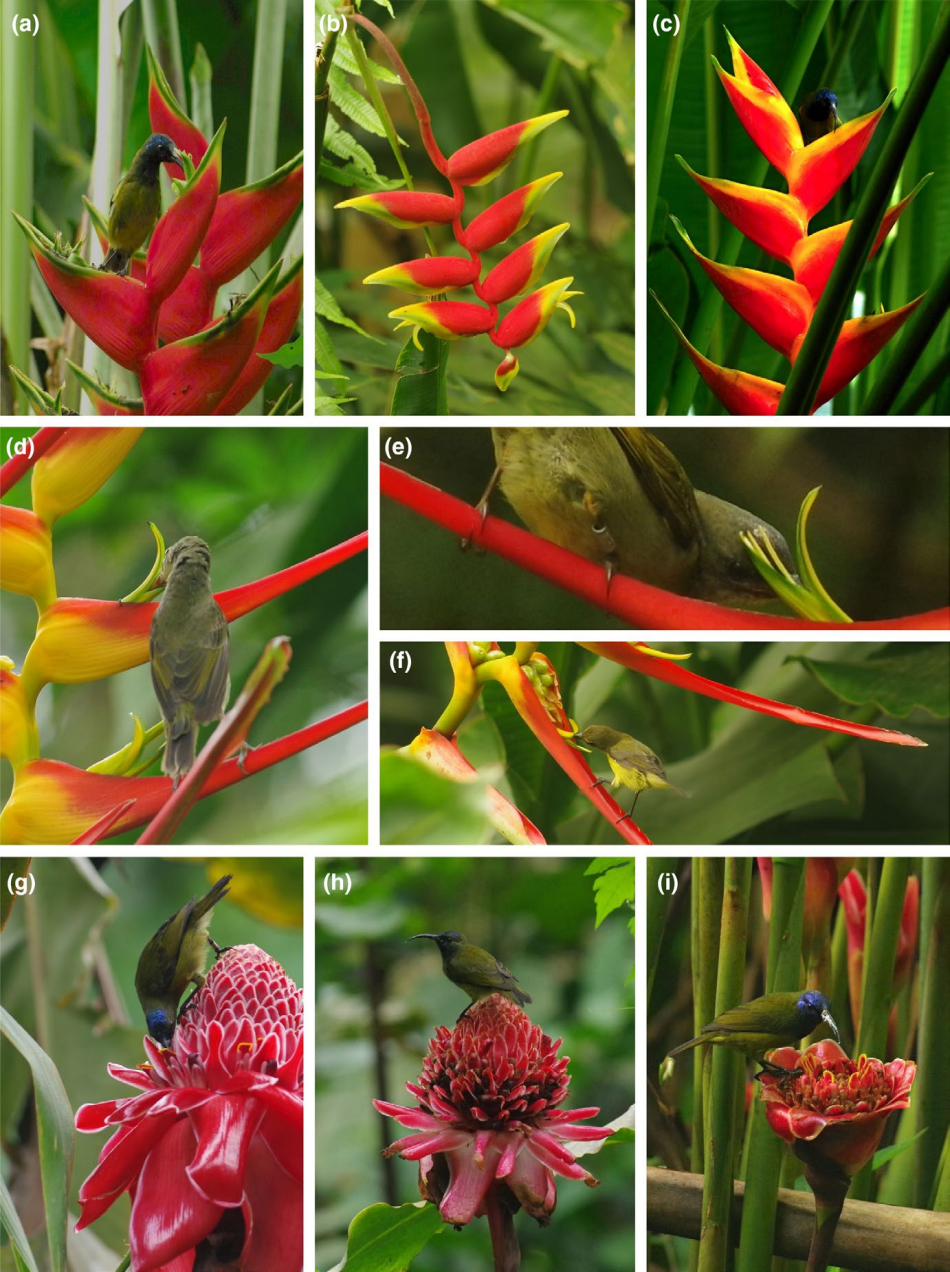
Studied plant and bird species. (a) *Cyanomitra oritis* on *Heliconia bihai*, (b) *Heliconia rostrata*, (c) *Heliconia caribaea* x *H. bihai* cv. Jacquinii, (d) and (e) *Cyanomitra olivacea* on *Heliconia latispatha* (f) female of *Cinnyris chloropygius* on *Heliconia latispatha* (g) *Cyanomitra oritis* on *Etlingera elatior* red form (with red invlolucral bracts), (h) *Cyanomitra oritis* on *Etlingera elatior* white form (with whitish‐pink involucral bracts), (i) *Cyanomitra oritis* on *Etlingera hemisphaerica*

All studied *Heliconia* spp. (Figure 1) are native to Central and/or South America. *H. bihai* (L.) is native in the area from Mexico to Brazil and Peru, it occurs also in the Caribbean. *H. latispatha* Benth. is native from East and South Mexico to NE Peru and Venezuela. *H. rostrata* Ruiz & Pavón is native from Mexico to Amazonian Peru and Ecuador (Andersson, 1981; Berry & Kress, 1991; Govaerts & Kress, 2019) and *H. bihai x caribaea* cv. Jacquinii is native in Grenada. In contrast to the genus *Heliconia,* genus *Etlingera* is native to Indo‐Pacific Asia. *Etlingera elatior* (Jack) R.M.Sm. is native in South Thailand, Malaysia, and Indonesia and *Etlingera hemisphaerica* (Blume) R.M.Sm. in Sumatra and Java (Choon & Ding, 2016; Govaerts & Newman, 2019). At the study site, there are two cultivated forms of *E. elatior* which represent the two extremes of the involucral bract color range occurring in nature: the red form with red involucral bracts and white form with whitish‐pink involucral bracts (Sabu & Smisha, 2013).

Both *Heliconia* and *Etlingera* spp. produce shoots bearing a maximum of one inflorescence. *Heliconia* spp. produce inflorescences almost always on the leafy shoots and in *Etlingera,* there are special shoots bearing either inflorescence or leaves. The individual flowers of both genera flower for a single day (Berry & Kress, 1991; Choon & Ding, 2016). In *Heliconia,* flowers are usually produced in red and/or yellow inflorescence bracts. Depending on the *Heliconia* species up to 50 flowers can be produced on one bract during the flowering period (Berry & Kress, 1991). Whole inflorescence can be either pendant (Figure 1b) or erect (Figure 1a,c,d). In the *Etlingera* genus, flowers are arranged in dense inflorescence heads containing alternating layers of floral bracts and flowers (Choon & Ding, 2016; Figure 1g–i). In *E. hemisphaerica,* the involucral bracts are red whereas in *E. elatior,* the involucral bracts can range from red (red form) to whitish‐pink (grown as white form in our study area) (Sabu & Smisha, 2013).

Most of the *Heliconia* species seem to be self‐compatible (Kress, 1983) as was shown also for *H. latispatha* (Kress, 1983) and *H. bihai* (Meléndez‐Ackerman, Rojas‐Sandoval, & Planas, 2008). Considering the genus *Etlingera,* it was shown that *E. elatior* is self‐compatible but self‐pollination is much less effective than cross pollination (Sabu & Smisha, 2013).

### 2.3 Plant traits

To compare the nectar value of individual species, we measured nectar production over 12 hr. The day before the nectar measurements, we marked flower buds and covered them with dense nets to prevent visitors from consuming the nectar after the flower had opened. If flowers opened on the day of nectar measurement, we marked them again as experimental flowers in the morning (around 6 a.m.). After 12 hr, we collected and measured nectar volume and concentration. Nectar volume was measured by Hamilton syringe (model 702 N) and concentration by pocket refractometer (PAL‐1, Atago Co.). Comparison of nectar production was performed on two days, on the 25 and 28 May 2018. The opening of the flowers was sometimes unpredictable and in consequence, we measured in total: 14 flowers on 10 plants of *E. elatior* red form; 11 flowers on 6 plants of *E. elatior* white form, 17 flowers on 10 plants of *E. hemisphaerica*, 9 flowers on 8 plants of *H. bihai*, 10 flowers on 10 plants of *H. bihai x caribaea* cv. Jacquinii; 9 flowers on 9 plants of *H. latispatha*, and 10 flowers on 10 plants of *H. rostrata*.

To compare nectar standing crop (i.e., actual amount of nectar under natural competition), we collected nectar twice per day from five nonmanipulated flowers, each of them on a different flowering shoot. The first harvest was around 10 a.m. and the second around 16 p.m. We measured nectar volume using a Hamilton syringe. Measurement of nectar standing crop was performed on two days 1st and 2nd June. Nevertheless, because of heavy rain on the 1st June in the afternoon, we measured only morning values on this date.

### 2.4 Sunbird visitors

Observations of visitors were performed on 11 days between 14 May 2018 and 2 July 2018. During each day, we observed one plant (flowering shoot) of each species. Observation was performed using AEE MagiCam 70S (AEE Technology Co., Ltd) sport cameras. Cameras were permanently connected to power banks. The duration of observations of individual plants on individual days differed due to logistical issues (charging of power banks, downloading data etc.) and some technical errors. In total, each species was observed approximately for 90 hr (Table S1). From the acquired video material, we extracted information on frequencies of visits per inflorescence, per flower and frequency of flower visits when the visitor was in contact with plant reproductive organs.

### 2.5 Experiment on sunbird pollination

To assess the significance of sunbirds in plant pollination, we set up a manipulative experiment. In this experiment, we covered randomly selected inflorescences with a sparse net with mesh size of 1 cm to exclude bird visitors. As a control, we marked nonmanipulated inflorescences and allowed them to be naturally exposed to visitors. The day after in the morning, when flowering was over, we collected gynoecia and fixed them in 96% ethanol for future analyses.

In the laboratory, we soaked the gynoecia in distilled water for 24 hr. and then put them into 10 M NaOH for 24 hr. Thereafter, we carefully washed the gynoecia in distilled water and left them overnight in aniline blue dye (Dafni, Kevan, & Husband, 2005). Pollen tubes were counted under a fluorescence microscope. Styles from one inflorescence collected on the same day represented one sample. The experiment took place from 18 till 24 May 2018.

To test whether there are any problems with pollen tube germination and/or staining (e.g., incompatibility of local pollens, problems with pollen tube coloring etc.), we also set up a hand‐pollinated control. In this subexperiment, we supplied stigmas of all species with pollen from distant flowering shoots in the morning. These gynoecia were then processed in the same way as those from the bird exclusion experiment.

### 2.6 Statistical analyses

Data from most of the datasets were not normally distributed, and therefore, we used analogical nonparametric permutation methods included in the PERMANOVA program which is an extension of the software PRIMER (Anderson, Gorley, & Clarke, 2008). Data on nectar volumes, visitation frequencies, number of pollen tubes were log (*x*+1) transformed to decrease the effect of extreme values of dependent variables. In the analyses on differences between genera, we treated species ID nested in Genus and Date (except for analysis on flower length) as random factors. When comparing species, we used Date as a random factor. In analyses on nectar production and concentration, when more samples were taken from one plant, individual plant ID was also treated as a random factor nested in plant species ID.

## 3 RESULTS

### 3.1 Plant traits

The studied plants differ in total flower length (Figure S1). *Heliconia* spp. have longer flowers than *Etlingera* spp.*,* but this difference was only marginally significant (perm. ANOVA, Genus: *F*_1,63_ = 7.86, *p* = .0557). Nevertheless, according to the video analysis the total flower lengths differ from functional lengths, that is terminal parts of flowers are relatively open which allows birds to enter deeper for nectar (the bird's reach is increased beyond the range of its bill and tongue). Flowers of genus *Etlingera* are hidden in compact inflorescences, and birds need to enter the flowers legitimately. In the genus *Heliconia,* flowers are more accessible and birds are able to insert their beak between the not fully united petals and sepals or even pierce the perianth without touching the reproductive organs (Figure 1f, Video [Link], [Link], [Link]).

**Figure 2:**
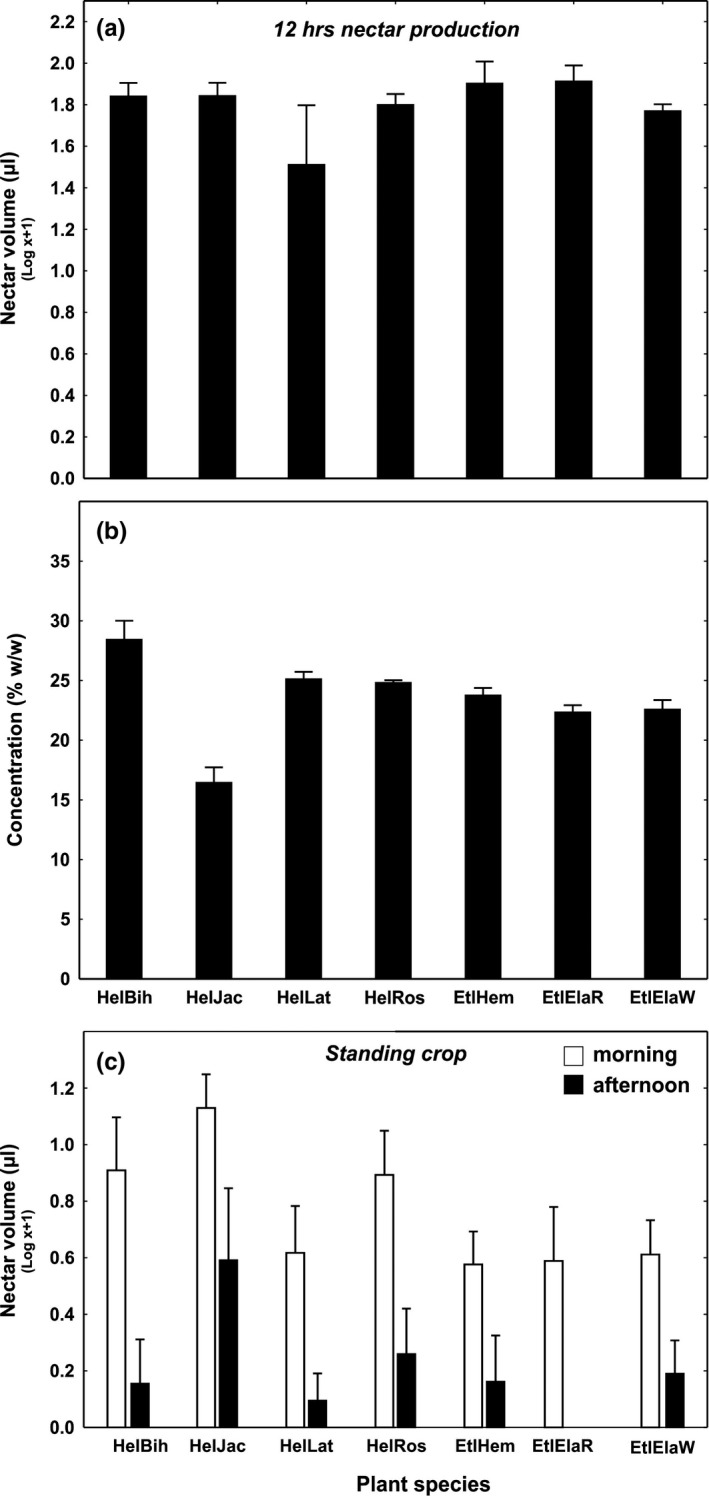
Nectar properties of studied plants. (a) 12 hr. nectar production in covered flowers, (b) concentration of nectar, (c) nectar standing crop in nonmanipulated flowers. *HelBih*, *Heliconia bihai*; *HelJac*, *Heliconia bihai* x *H.caribea* (”Jacquinii”); *HelLat*, *Heliconia latispatha*; *HelRos*, *Heliconia rostrata*; *EtlHem*, *Etlingera hemisphaerica*; *EtlElaR*, *Etlingera elatior* red form; *EtlElaW*, *Etlingera elatior* white form. Means plus SE are shown

Studied plants did not differ in nectar production per flower over 12 hr. (mean = 80.9 μl ± 8.6 SE, Figure 2a; perm. mixed‐effect model; Plant Species: *F*_6,39_ = 0.96; *p* = .4726). However, nectar concentration differed between species (Figure 2b; perm. mixed‐effect model; Plant Species: *F*_6,39_ = 10.99, *p* = .0001). No significant difference was found between *Heliconia* and *Etlingera* genus in nectar concentration (perm. mixed‐effect model; Genus: *F*_1,39_ = 0.05; *p* = .8420). Nectar standing crop differed among species and at different harvest times (morning vs. afternoon), the diurnal changes (interaction) nevertheless did not differ among species (Figure 2c; perm. mixed‐effect model; Plant Species: *F*_6,90_ = 2.71, *p* = .0202; Harvest time: *F*_1,90_ = 15.29, *p* = .0001; Interaction Plant Species x Harvest time: *F*_6,90_ = 0.26; *p* = .9532). When considering genera, *Etlingera* had lower standing crops, but this difference was only marginally significant. There was no significant difference between genera in standing crop diurnal changes (perm. mixed‐effect model; Genus: *F*_1,95_ = 3.14; *p* = .0873; Harvest time *F*_1,95_ = 14.96; *p* = .0002; Interaction Genera x Harvest time *F*_1,95_ = 0.61; *p* = .4331).

### 3.2 Sunbird visitors

Studied plant species were visited by three sunbird species: *Cinnyris chloropygius*, *Cyanomitra olivacea,* and *Cyanomitra oritis*. Although the cameras were set up to record sunbirds (i.e., cameras were as far as possible from the plants to avoid scaring bird visitors, and each inflorescence was recorded from just one side), we also observed some insect visitors. The most common were honeybees but also butterflies and ants were observed on both *Heliconia* and *Etlingera* spp.

The most frequently visited species were *E. elatior* white form (0.94 bird visits flower^−1^ hr^−1^), *E. hemisphaerica* (0.91), *E. elatior* red form (0.89), and *H. latispatha* with 0.88 bird visits flower^−1^ hr^−1^. The other three *Heliconia* spp. were visited much less often. *H. bihai* had a visitation frequency of 0.20 bird visits flower^−1^ hr^−1^, *H. bihai x caribaea* cv. Jacquinii 0.1 visits and *H. rostrata* only 0.07 visits flower^−1^ hr^−1^.

**Figure 3:**
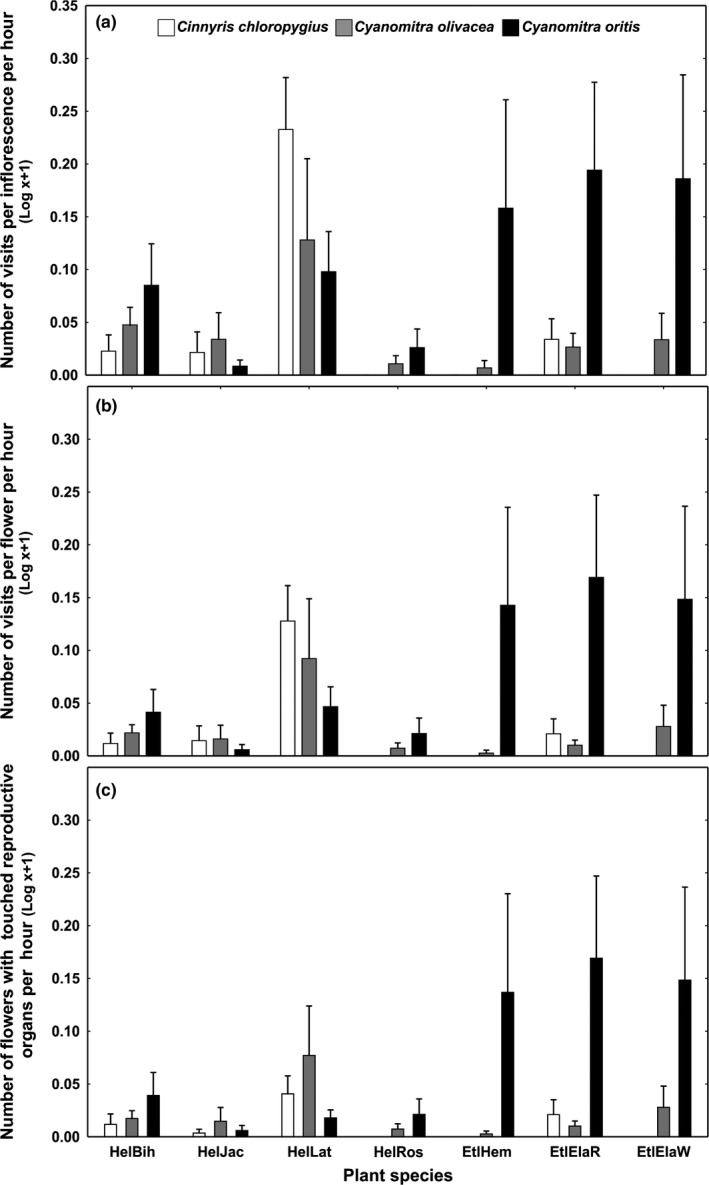
Frequencies of visits on (a) inflorescences, (b) flowers, and (c) flowers when reproductive organs were touched. For plant abbreviations, see Figure 2. Means plus SE are shown

Both individual plant species and plant genera differed in the spectrum of bird visitors, regardless of whether the frequency of visits per inflorescence or per flower is considered (Table 1). *Etlingera* species were visited mainly by *C. oritis*., *H. latispatha* was visited mainly by *C. chloropygius* followed by *C. olivacea* and *C. oritis*. A much higher frequency of visits to *H. latispatha* was recorded per inflorescence than per flower, and this difference was more obvious than for *Etlingera* species (Figure 3a,b). When we tested the opposite, that is how bird species differed in the spectrum of visited plants, there were significant differences between bird species when considering frequency of visits per inflorescence but only a marginally significant difference when considering frequency per flower (Table 1; Figure S2).

**Table 1:** PERMANOVA analyses on differences between visitor communities on plant species (i.e., plant species as an explanatory variable), differences in visitor communities between genera (i.e., plant genera as an explanatory variable), and differences in the spectrum of plants visited by individual bird species (i.e., bird species as an explanatory variable) and comparing visitation community on individual plant species (i.e., plant species as an explanatory variable). Data were log (*x*+1) transformed. Date was used as a random effect in all analyses. In addition, to test the effect of genus we considered plant species nested in factor genus as a random factor

	Plant species		Plant genera		Bird species	
	*F*_ps_	*p*	*F*_ps_	*p*	*F*_ps_	*p*
Frequency of visits per inflorescence	4.50	.0001	3.35	.0284	3.12	.0293
Frequency of visits per flower	3.31	.0005	8.36	.0278	2.81	.0601
Frequency of touching reproductive organs	2.73	.0071	25.26	.0287	2.68	.0720

When considering sunbird pollination potential (expressed as frequency of contacts with reproductive organs), sunbird pollination communities of individual plants and genera differed (Table 1; Figure 3c). Contact with reproductive organs was detected, due to the structure of flowers, during all visits on *Etlingera* species (Figure 1g) and the beaks of sunbirds were often densely covered with sticky pollen grains (Figure 1i; Video S3). In contrast, on the most often visited *Heliconia*, *H. latispatha,* sunbirds frequently drank nectar without touching the reproductive organs (Figure 1f; Video S1). In *H. latispatha,* 16.6% of flower visits of *C. chloropygius*, 89.2% visits of *C. olivacea,* and 39.5% visits of *C. oritis* involved contact with reproductive organs. The analysis of the opposite scenario (i.e., if individual bird species differ in the spectrum of plant species of which they were in contact with reproductive organs) showed only marginally significant results (Table 1; Figure S2c).

### 3.3 Experiment on sunbird pollination

**Figure 4:**
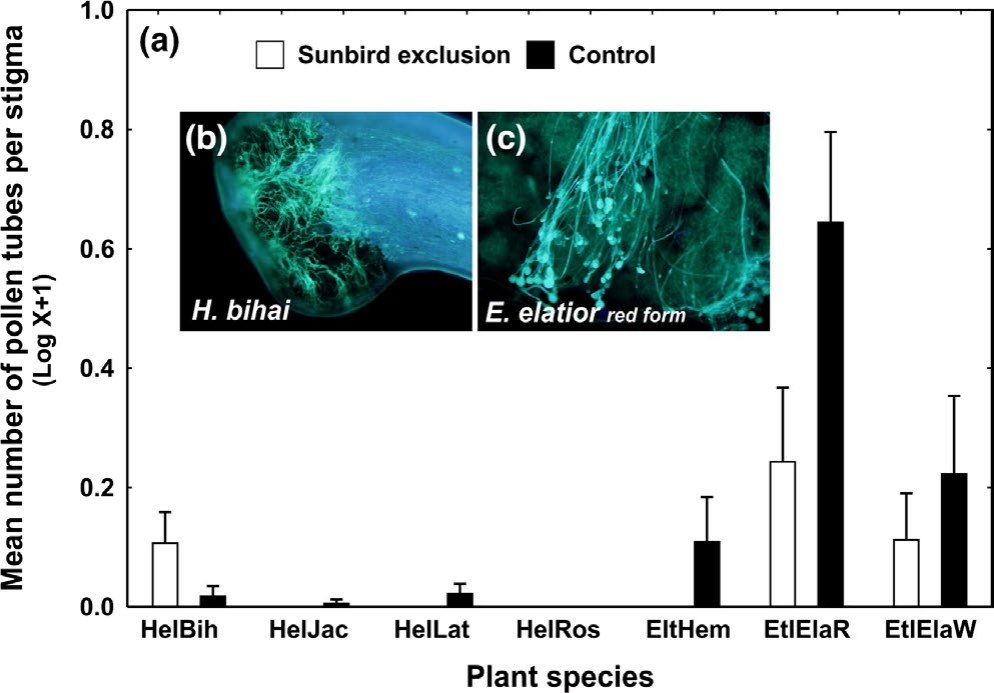
(a) Results of experimental exclusion of sunbirds. Germinating pollen grains on stigma of *Heliconia bihai* (b) and *Etlingera elatio*r (c). For plant abbreviations, see Figure 2. Means plus SE are shown

After a controlled hand pollination supplement, we observed germinating pollen grains on the stigmas of *Etlingera* spp. (Figure 4c). In contrast, almost no germinating pollen grains were observed on the stigmas of *Heliconia* spp. (Figure S3). The same pattern was observed in the experiment (Figure 4). Pollen tubes on *Heliconia* plants moreover germinated on the stigma and did not grow into the style (Figure 4b). Plant species differed in pollen tube number, sunbird exclusion had a negative effect on the number of pollen tubes, and this treatment was species‐specific (perm. mixed‐effect model; Plant: *F*_6,334_ = 11.81, *p* = .0001; Treatment: *F*_1,334_ = 6.90, *p* = .0092; Plant x Treatment *F*_6,334_, *p* = .0020). Genera differed in the number of pollen tubes and were differently affected by the treatment (perm. mixed‐effect model; Genus: *F*_1,339_ = 4.61, *p* = .0292; Treatment: *F*_1,339_ = 9.46; Genus x Treatment *F*_1,339_ = 12.43; *p* = .0006).

## 4 DISCUSSION

Our study demonstrated a high degree of ecological fitting. We also show that distant evolutionary history of individual actors cannot prevent fundamental ecological processes such as occupation of new niches by alien plants or niche partitioning.

Our study complements the ideas of Janzen (1985) that most of the interactions we see around us are not necessarily the consequences of coevolutionary processes in a given place, but of simple ecological fitting. Janzen´s main arguments were that biological communities are dominated by widespread invasive species, which have extended their range and are not originally adapted to most of their habitats. From this point of view, our study can be considered experimental support of this statement showing that organisms from three different continents can create a complex interacting community on the basis of ecological fitting.

However, we have also shown that the Asian *Etlingera* spp. established tighter interactions with African sunbirds. *Etlingera* spp. were visited legitimately more often, and sunbirds seem to be very effective pollinators. From the sunbirds’ point of view, it does not seem to be a simple consequence of bigger rewards, because both *Etlingera* and *Heliconia* spp. produced similar amounts of nectar. *Etlingera* spp. even had a slightly lower nectar standing crop (but only marginally significant). We assume the reason for this perfect ecological fit could be a more comfortable perching position when feeding and from the plant's point of view, the compact inflorescence enables only legitimate entering of the flowers. Pollen of *Etlingera* spp. is then precisely placed in high quantity on the bills of perching sunbirds. *Heliconia* spp. had weaker but still functional interactions with the local sunbirds which is amazing if we consider that the genus *Heliconia* is in the oldest known clade of hummingbird‐pollinated plants (Iles et al., 2017). We were surprised how *H. rostrata,* which is pollinated by hovering hummingbirds (Iles et al., 2017), is able to precisely place pollen on the heads of perching *C. olivacea* and *C. oritis* (Video S2). *Heliconia* spp. were not visited with the same frequency indicating that also intragenus trait differences are important. We assume that the high visitation rate of *H. latispatha* is mainly due to fact that the flowers are not deeply hidden in bracts and can be more easily reached in both legitimate and illegitimate ways. Although we observed frequent contacts of *Heliconia* reproductive organs and even pollen on the heads of sunbirds, we were not able to evaluate pollinator effectivity directly by counting germinated pollen tubes. There were almost no pollen tubes observed, or they did not grow inside the style. Because this was the case not only for experimental treatments but also for hand pollination, we cannot presume that this was the consequence of pollinator infectivity in pollen transport. In consequence, we can only speculate whether this was caused by intraspecific pollen transport or by high inbreeding depression because of low genetic diversity on the farms where plants are propagated only clonally. Although we optimized the staining technique over a long time period and followed experimental methods of other researchers working on *Heliconia* pollen tubes (Betts, Hadley, & Kress, 2015), it is possible that these negative results are the consequence of a methodological mistake. This can be supported by the fact that *Heliconias* on the farm produced fruits (including *H. rostrata* of which we did not observed any pollen tubes).

On the plantation, we also observed niche differentiation among individual plant and bird species. *Etlingera* spp., which are adapted to long‐billed spiderhunters in Asia (Sakai et al., 1999; 2013), were visited in the new habitat by long‐billed sunbirds *C. oritis*. The medium bill‐sized sunbird *C. olivacea* visited mainly *H. latispatha* with more reachable nectar but also fed on *Etlingera *spp. The short‐billed *C. chloropygius* visited mainly *H. latispatha* where it, however, mainly thieved nectar. *H. bihai*, which has flowers more hidden in the bracts, was mostly visited by long‐billed *C. oritis*. This type of niche differentiation based on bill length was described for two *Heliconia* and three hummingbird species in Costa Rica by Taylor and White (2007), but we can find it also in others natural communities both in the New (Feinsinger, Swarm, & Wolfe, 1985) and Old World (Ford & Paton, 1977; Geerts & Pauw, 2009b; Janeček et al., 2012). The thieving behavior of short‐billed birds on long tubular flowers, as observed mainly for *H. latispatha*–*C. chloropygius* interaction, is common in natural pollination systems. This behavior was shown for sunbirds (Geerts & Pauw, 2009b; Janeček et al., 2015) as well as for hummingbirds (Gill, 1987; Maglianesi, Blüthgen, Gaese, & Schleuning, 2014; Maruyama, Bugoni, Dalsgaard, Sazima, & Sazima, 2015).

Using this example of a semiarbitrary plant–bird community from three continents, we demonstrated that potential ornithophilous invasive plants can be easily incorporated into local communities. In consequence, we are delivering a similar message from Africa as Maruyama et al. (2016) from America. Sunbird pollination networks and hummingbird networks are open to exotic plant species. Nevertheless, it is a question to which degree this is a worrying message. From the birds’ point of view, local flower farms represent a rich nectar source. During our research on Mt. Cameroon, we never observed such a high density of sunbirds anywhere else in the region. For example, at the plantation with ornamental flowers we caught 62 individuals of the endemic sunbird *C. oritis* during three days of extensive mist‐netting, whereas at a congruent elevation in natural forest on Mt. Cameroon, we only caught 15 individuals of *C. oritis* despite a comparable amount of sampling effort. The effect on local flora can nevertheless be much more controversial. It was demonstrated that alien plants have a negative effect on both visitation and reproductive success of native coflowering species (Morales & Traveset, 2009). In consequence, the effects of these plantations on local flora need to be studied in detail.

## AUTHOR CONTRIBUTIONS

ŠJ conceived the ideas and designed methodology. Fieldwork and laboratory work was conducted by ŠJ, KC, GU, PJ, EC, ZS, and FLE. ŠJ analyzed the data. ŠJ led the writing of the manuscript. All authors contributed critically to the drafts and gave approval for publication.

## Data Availability

Data available from the Dryad Digital Repository: https://doi.org/10.5061/dryad.qnk98sfbx
