# Supplementary material for: Ecological fitting is a sufficient driver of tight interactions between sunbirds and ornithophilous plants
Source: Ecol Evol. 2020 Feb 5;10(4):1784–93. doi: 10.1002/ece3.5942 (PMC7042734; doi:10.1002/ece3.5942)
Supplement: Supplementary file 1 [file ECE3-10-1784-s001.docx]

**Table S1**. Sampling effort. Duration of observations of individual plants (hours). ***HelBih*** - *Heliconia bihai*, ***HelJac*** = *Heliconia bihai* x *H.caribea* (”Jacquinii”), ***HelLat*** - *Heliconia latispatha* , ***HelRos*** - *Heliconia rostrata*, ***EtlHem*** - *Etlingera hemisphaerica*, ***EtlElaR*** - *Etlingera elatior* red form, ***EtlElaW*** - *Etlingera elatior* white form

| **Plant No.** | **HelBih** | **HelJac** | **HelLat** | **HelRos** | **EtlHem** | **EtlElaR** | **EtlElaW** |
| --- | --- | --- | --- | --- | --- | --- | --- |
| **1** | 5.5 | 5.5 | 5.6 | 5.7 | 5.4 | 5.2 | 4.7 |
| **2** | 6.4 | 5.5 | 5.5 | 5.5 | 6.6 | 5.2 | 4.7 |
| **3** | 11.5 | 10.5 | 11.7 | 12 | 11 | 9.6 | 11.8 |
| **4** | 5.5 | 5.9 | 10.4 | 5.3 | 5.8 | 6 | 5.8 |
| **5** | 8.6 | 7.3 | 10.4 | 10.9 | 9.1 | 10.5 | 10.7 |
| **6** | 10.4 | 10.4 | 10.6 | 10.5 | 9.3 | 10.6 | 10.4 |
| **7** | 10.1 | 10.7 | 10.2 | 10.3 | 10.4 | 10.5 | 9.1 |
| **8** | 6.5 | 7 | 7.1 | 6.9 | 7 | 6.9 | 2.3 |
| **9** | 6.2 | 7.8 | 6.1 | 5.7 | 6.2 | 5.9 | 6.1 |
| **10** | 10.5 | 1.9 | 10.7 | 10.2 | 10.5 | 10.2 | 10.8 |
| **11** | 10.6 | 8.5 | 10.6 | 10.2 | 10.4 | 9.2 | 10.8 |
| **TOTAL** | **91.8** | **81** | **98.9** | **93.2** | **91.7** | **89.8** | **87.2** |

**
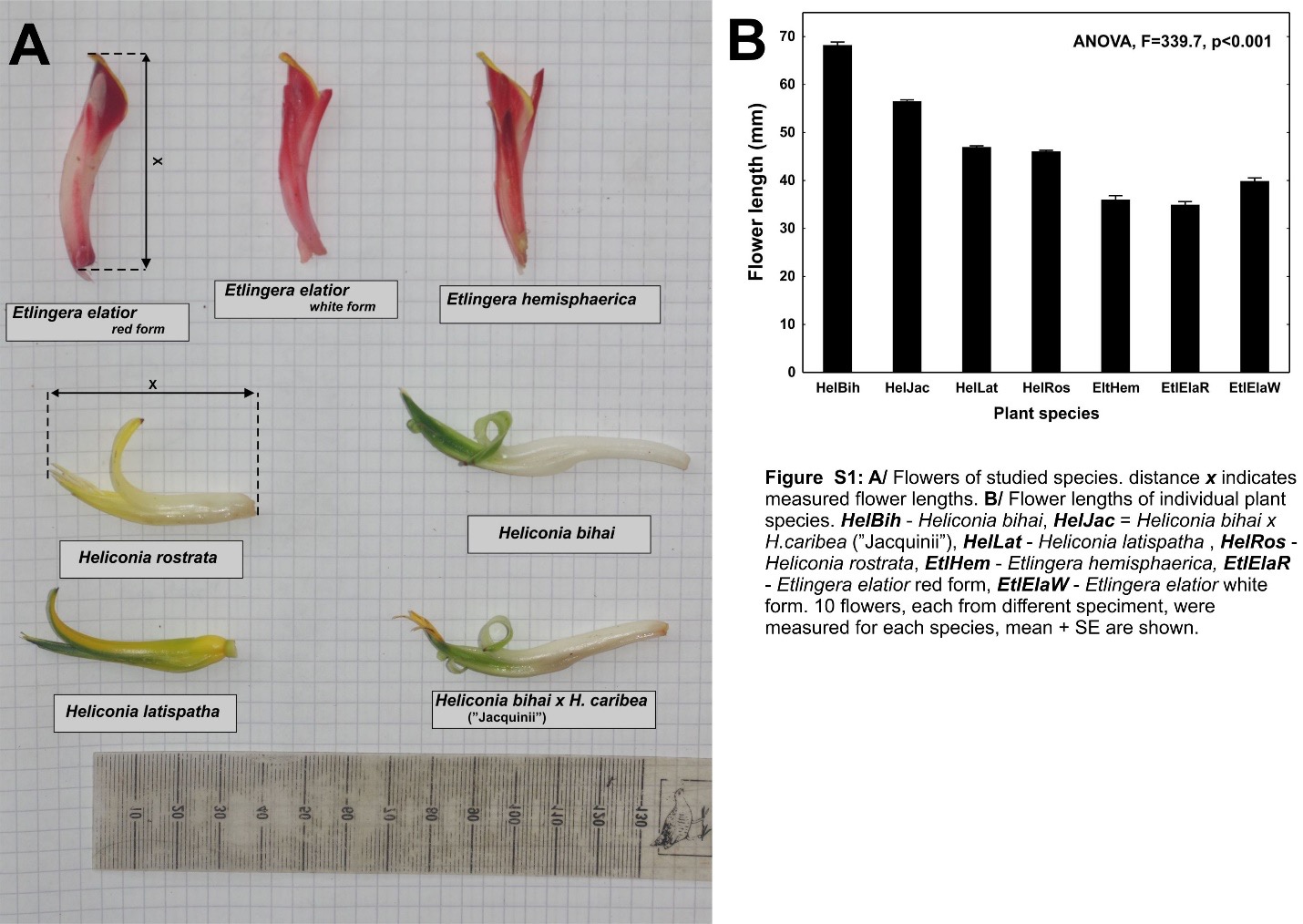
**

**Figure S1**

**
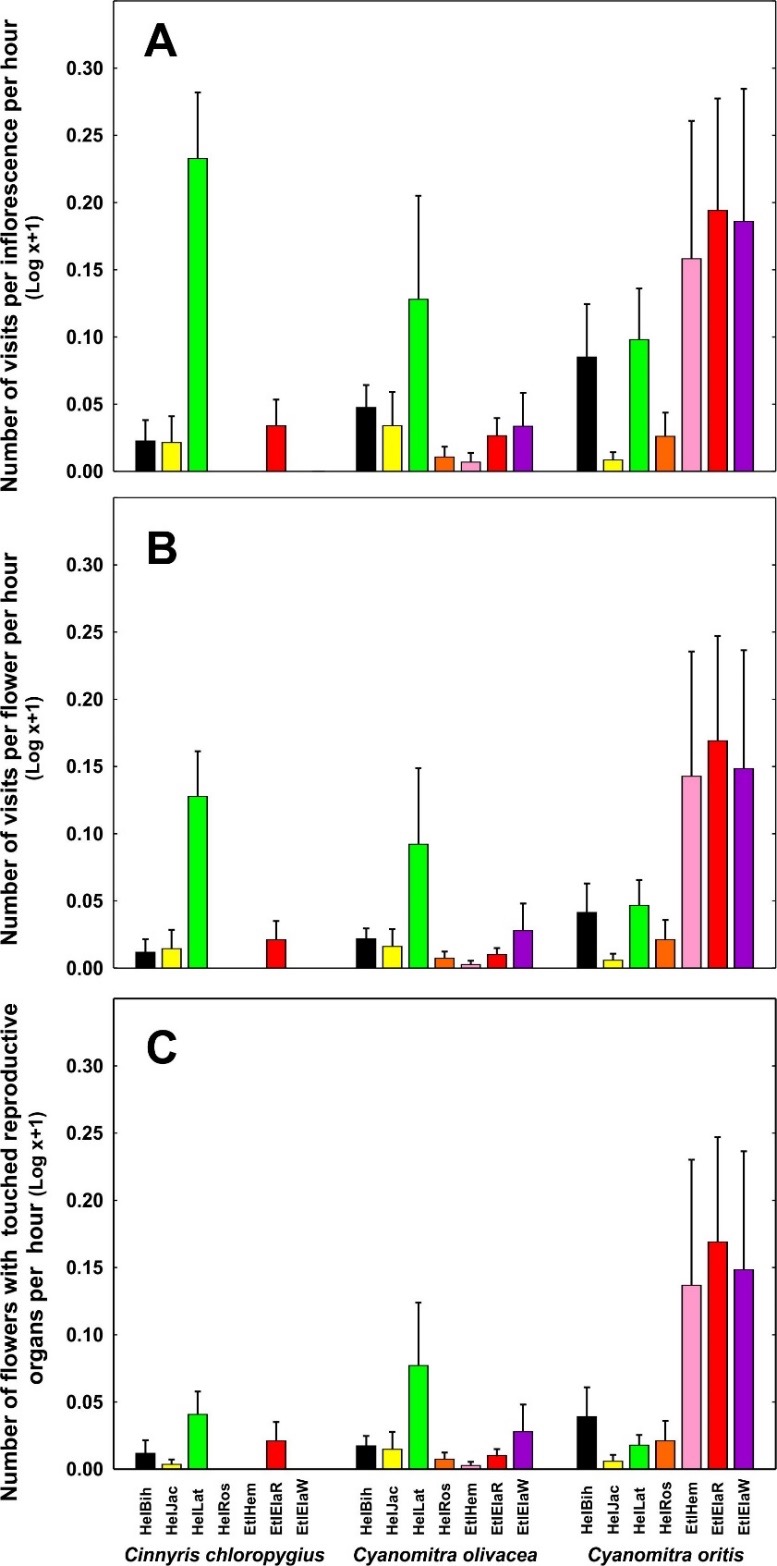
**

**Figure S2:** Frequencies of visits on **A/** inflorescences, **B/** flowers and **C/** flowers with touched reproductive organs. ***HelBih*** - *Heliconia bihai*, ***HelJac*** = *Heliconia bihai* x *H.caribea* (”Jacquinii”), ***HelLat*** - *Heliconia latispatha* , ***HelRos*** - *Heliconia rostrata*, ***EtlHem*** - *Etlingera hemisphaerica*, ***EtlElaR*** - *Etlingera elatior* red form, ***EtlElaW*** - *Etlingera elatior* white form. Means plus SE are shown.

**
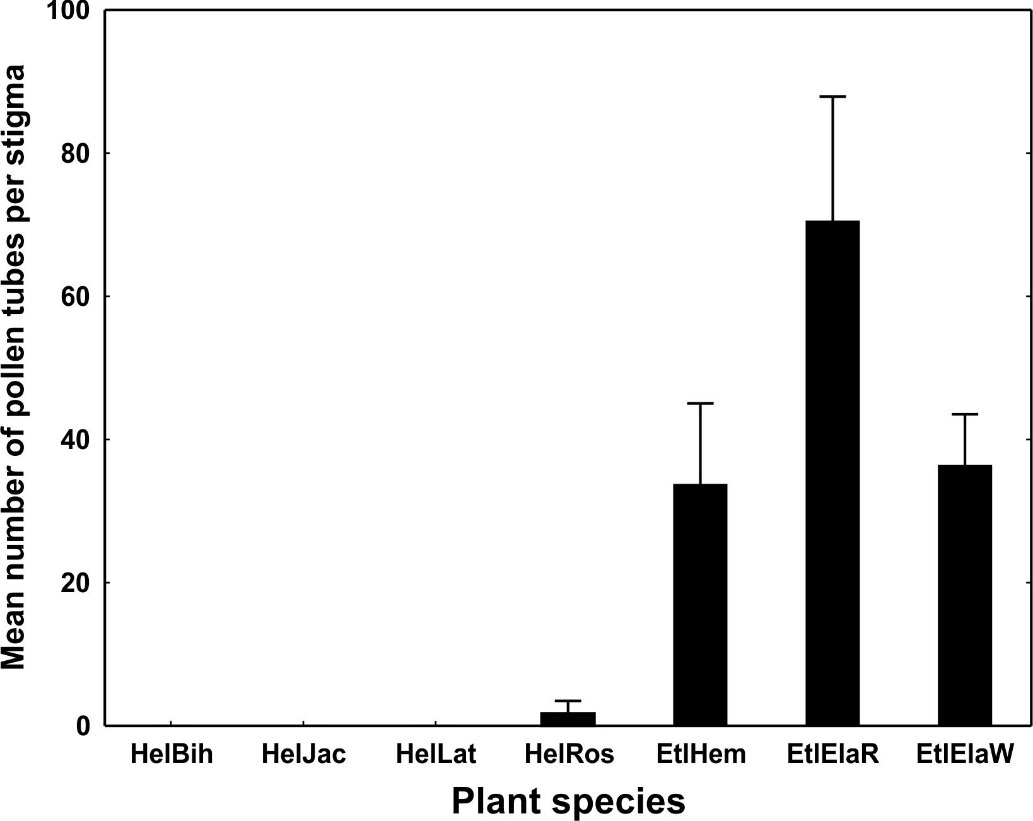
**

**Figure S3:** Hand pollination of studied plants. ***HelBih*** - *Heliconia bihai*, ***HelJac*** = *Heliconia bihai* x *H.caribea* (”Jacquinii”), ***HelLat*** - *Heliconia latispatha* , ***HelRos*** - *Heliconia rostrata*, ***EtlHem*** - *Etlingera hemisphaerica*, ***EtlElaR*** - *Etlingera elatior* red form, ***EtlElaW*** - *Etlingera elatior* white form. Means plus SE are shown.
